# Homo-dimerization and ligand binding by the leucine-rich repeat domain at *RHG1/RFS2* underlying resistance to two soybean pathogens

**DOI:** 10.1186/1471-2229-13-43

**Published:** 2013-03-15

**Authors:** Ahmed J Afzal, Ali Srour, Abhishek Goil, Sheeja Vasudaven, Tianyun Liu, Ram Samudrala, Navneet Dogra, Punit Kohli, Ayan Malakar, David A Lightfoot

**Affiliations:** 1 Department of Molecular Biology, Microbiology and Biochemistry and Center for Excellence the Illinois Soybean Center, Southern Illinois University at Carbondale, IL 62901, USA; 2 Genomics Core Facility; Department of Plant Soil and Agricultural Systems, Southern Illinois University at Carbondale, Carbondale, IL 62901-4415, USA; 3 Department of Molecular Biology and Biochemistry, University of California at Irvine, Irvine, CA 92697-4560, USA; 4 Department of Microbiology Box 357242, University of Washington, Seattle, WA 98195-7242, USA; 5 Department of Chemistry, Southern Illinois University at Carbondale, IL 62901, USA

**Keywords:** Receptor, Leucine-rich repeat, Ligand, Peptide, Cross-link, Predicted

## Abstract

**Background:**

The protein encoded by GmRLK18-1 (*Glyma_18_02680* on chromosome 18) was a receptor like kinase (RLK) encoded within the soybean (Glycine max L. Merr.) *Rhg1/Rfs2* locus. The locus underlies resistance to the soybean cyst nematode (SCN) *Heterodera glycines* (I.) and causal agent of sudden death syndrome (SDS) *Fusarium virguliforme* (Aoki). Previously the leucine rich repeat (LRR) domain was expressed in *Escherichia coli*.

**Results:**

The aims here were to evaluate the LRRs ability to; homo-dimerize; bind larger proteins; and bind to small peptides. Western analysis suggested homo-dimers could form after protein extraction from roots. The purified LRR domain, from residue 131–485, was seen to form a mixture of monomers and homo-dimers *in vitro*. Cross-linking experiments *in vitro* showed the H274N region was close (<11.1 A) to the highly conserved cysteine residue C196 on the second homo-dimer subunit. Binding constants of 20–142 nM for peptides found in plant and nematode secretions were found. Effects on plant phenotypes including wilting, stem bending and resistance to infection by SCN were observed when roots were treated with 50 pM of the peptides. Far-Western analyses followed by MS showed methionine synthase and cyclophilin bound strongly to the LRR domain. A second LRR from GmRLK08-1 (Glyma_08_g11350) did not show these strong interactions.

**Conclusions:**

The LRR domain of the GmRLK18-1 protein formed both a monomer and a homo-dimer. The LRR domain bound avidly to 4 different CLE peptides, a cyclophilin and a methionine synthase. The CLE peptides GmTGIF, GmCLE34, GmCLE3 and HgCLE were previously reported to be involved in root growth inhibition but here GmTGIF and HgCLE were shown to alter stem morphology and resistance to SCN. One of several models from homology and *ab-initio* modeling was partially validated by cross-linking. The effect of the 3 amino acid replacements present among RLK allotypes, A87V, Q115K and H274N were predicted to alter domain stability and function. Therefore, the LRR domain of GmRLK18-1 might underlie both root development and disease resistance in soybean and provide an avenue to develop new variants and ligands that might promote reduced losses to SCN.

## Background

Plants employ both cell surface and cytoplasmic receptors to respond to a wide array of signals from pathogens [1]. The receptor protein kinases (RPKs) represent one of the two large gene families implicated to underlie the recognition events that lead to pathogen resistance [2]. Two of the most destructive pathogens in soybean (*Glycine max* L. Merr*.*) are the soybean cyst nematode (SCN; *Heterodera glycines* I.) and sudden death syndrome (SDS) agent *Fusarium virguliforme* (Aoki) [3]. The complex genetics of the cyst nematode populations, the partial nature of plant resistance and temperature sensitivity makes controlling the nematode a difficult task [4,5]. Elicitation of plant defenses in response to the pathogens were shown to involve the activity of RLK proteins [5-8] introgressed from Peking. Two loci, *Rhg4* on chromosome 8 (linkage group (Lg) A2) and *Rhg1/Rfs2* on chromosome 18 (Lg G), contain genes that encode receptor like kinase (RLK) proteins within the RPK gene family implicated in resistance. GmRLK08-1 (Glyma_08_11350) is near *Rhg4* and GmRLK18-1 (Glyma_18_02680) is within *Rhg1/Rfs2*[5,8-10]. The translated proteins are both RLKs with extra-cellular leucine rich repeats (LRR). Many other genes can alter SCN responses. However, only GmRLK18-1 has been shown to underlie resistance to both pathogens in transgenic plants [8,9]

Numerous studies have implicated the LRR domain of RLKs in effector recognition and protein-protein interactions [1,2,5,11-19]. The intracellular kinase domains of RLKs are often involved in phosphorylation mediated signal transduction. The GmRLK18-1 protein shows separated domains of different function that are a characteristic feature of both plant and animal RLKs. The extracellular domains are predicted to be involved in dimerization/recognition and the intracellular domain is involved in signal transduction. The GmRLK18-1 protein was predicted to be an 855 amino acid polypeptide that encoded an N-terminal signal peptide (amino acids 1–61), 10 extracellular leucine rich repeats (amino acids 141–471), a single pass trans-membrane domain (amino acids 485–507), and an intracellular serine/threonine kinase domain (amino acids 510–855; 5).

At *Rhg1/Rfs2,* the resistance phenotypes were perfectly associated with the GmRLK18-1 allotype 1 [5] and that allele in transgenic plants provided partial resistance [8]. Combined, the amino acid changes (A87V, Q115K and H274N) were sufficient to differentiate between plant introductions possessing type I resistance (Peking based resistance) and four other allotypes. No studies to date have attempted to analyze the role of these amino acid changes on overall protein structure, hence the molecular basis of resistance to SCN and SDS pathogenesis remains unexplored.

A recent study [6] shed light on secondary structural components of the GmRLK18-1 LRR domain. Helix and sheet content coincided with an alpha beta structural fold. Some unstructured elements within the LRR domain were inferred through circular dichroism (CD) spectrometry. Allotype comparisons were not yet made due to inherent refolding problems associated with some LRR proteins. In many instances, protein structure can be predicted by comparison to homologs of known structure [20-23]. For the GmRLK18-1 LRR-domain residues 141–435 expressed in *E. coli*[6] the nearest orthologs, judged by primary sequence similarity and length, with known structures included; 1ogq_a (PGIP) [16], 3rgx (BRI1) [18,19], 1xcd_a (decorin) [21], 1o6s (E-cadherin) [15]; 1ozn_a (NOGO receptor, ligand binding domain) [22]; and 2bnh (porcine ribonuclease inhibitor; PRI) [23].

Disregarding the length of the LRR domain, the polygalacturonase-inhibiting protein from *Phaseolus vulgaris* (PGIP) [16] was the closest ortholog of GmRLK18-1 with a known structure, sharing 27 percent identity and 44 percent similarity (http://www.sbg.bio.ic.ac.uk/~phyre2/html/page.cgi?id=index). Next was the BRI1 receptor [18,19] that was 27% identical and 42% similar in the LRR region (residues 141–435). The PRI protein [23] shared 20% identity and 36% similarity with the GmRLK18-1-LRR, was of similar length and was known to form homo-dimers *in vivo*. Among well studied plant RLK-R proteins the LRR of GmRLK18-1 was most similar (39%) to the rice XA21 receptor kinase LRR [11,24]. XA21 is a RLK, the extra-cellular domain of which controls race specific pathogen recognition in response to a known elicitor. The binding of the pathogen ligand to the XA21 LRR-domain may result in dimerization and activation of intracellular kinase. Among genes involved in the control of development the GmRLK18-1 LRR domain was 43% similar to Arabidopsis CLAVATA1 and CLAVATA2 that heterodimerize [25]. The GmRLK18-1 LRR domain was 45% similar to soybean NARK1 [26]. These latter 3 proteins have been shown to bind CLE peptides as part of their activity. There is a CLE peptide in nematode secretions that was shown to be perceived by RLKs in the *CLAVATA2* and *CORYNE* families [25] raising the possibility that GmRLK18-1 might bind CLE peptides. Binding constants (Kd) for CLE peptides were reported in the range of 17.4-2,000 nM.

Previously a three dimensional model for an RPK protein [27] was predicted but the modeled RPK protein was not an RLK. Equally, a model for and RLK was predicted, but the protein acted in symbiosis not defense [26]. Here a model of the LRR domain from a RLK protein involved in resistance is reported based on homology modeling of the extracellular LRR domain (residue 141–471) of the GmRLK18-1 protein. Modeling for GmRLK18-1-LRR was based on PRI (2BNH) [23]. The effect of the amino acid substitutions on protein stability was inferred; structure for fold and class analysis was made; and secondary structure was analyzed *in vitro* and *in silico* to predict whether GmRLK18-1 may homo-dimerize *in vivo*. The models were tested with proteins cross linked *in vitro*. Ligand binding was measured with short CLE-like peptides.

## Results

### Analyses of protein sequences

**Figure 1:**
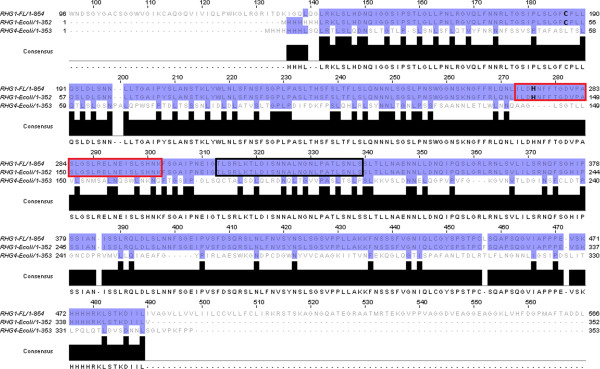
**Sequences of the GmRLK18-1 protein and the LRR domains expressed in *E. coli*.** The whole RLK protein theoretical pI was 8.42 and the molecular weight was 92,388.98 Da. B. LRR domain fragment expressed in *E. coli*. Boxed was the peptide used to raise a specific antibody. In bold are the cysteine residue labeled by cross linking and the histidine residue polymorphic in resistant and susceptible plants. Boxed red is the trypsin fragment in contact with the cysteine when the homo-dimer forms; note it contains the histidine residue. The protein predicted pI was 9.54 and molecular weight 38,404.55 Da. Also shown was the amino acid sequence of the LRR domain of GmRLK08-1 near *Rhg4* that was expressed in *E. coli* and used for ligand binding assays. The proteins predicted pI was 5.2 and molecular weight 38,086.11 Da. The LRR domain showed 45% similarity with that of GmRLK18-1.

The GmRLK18-1-LRR domain showed diverged motifs but in a regularly repeating pattern (Figure 1) [2]. Conversely, the kinase domain contained the expected conserved motifs and therefore appeared to have experienced purifying selection. The synonymous and non-synonymous substitution rates in both these domains differed as well (Figure 1). Eight of the nine known alleles of the RLK at the *rhg1* locus [5] were aligned using CLUSTAL-W and rates of synonymous and non-synonymous substitutions determined. The ratio of non-synonymous to synonymous substitutions in the LRR-domain was observed to be about 1:1 whereas in the kinase domain, most nucleotide substitutions did not translate into amino acid changes and the ratio exceeded 3:1. The ratios suggest purifying selection eliminated mutants in the kinase domain but not the LRR domain. The relative lack of amino acid sequence variance per nucleotide change may be associated with the role of the kinase domain in the signaling function. Appropriate message transduction imposes severe constraints on amino acid substitutions. In contrast, the LRR-domains may be expected to accumulate mutations that are neutral, improve the current function or lead to new adaptive recognition capabilities.

### Detection of homo-dimers for GmRLK18-1 but not GmRLK08-1

**Figure 2:**
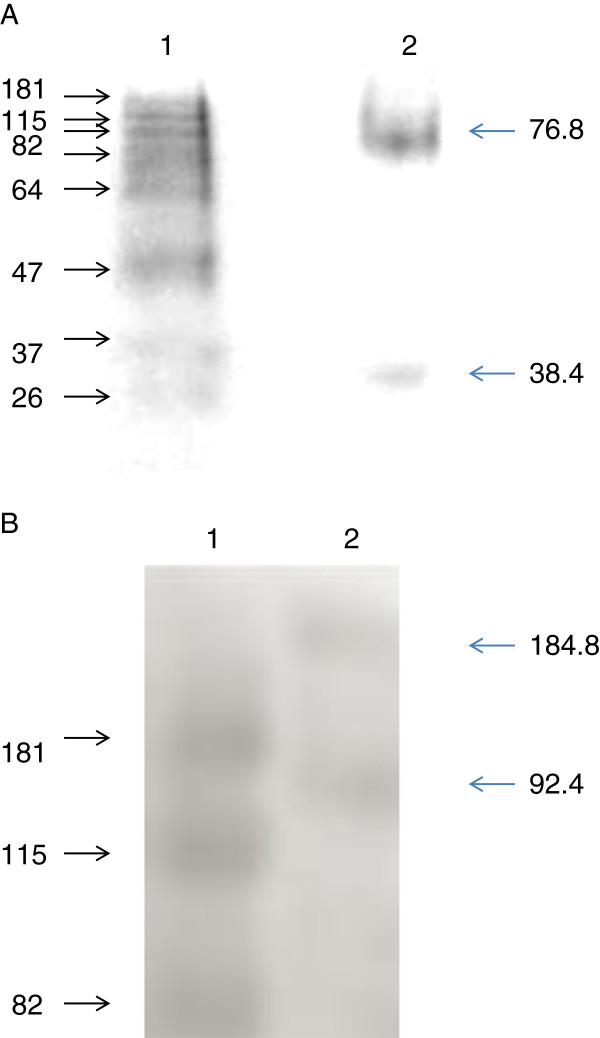
**Evidence for dimerization by the GmRLK18-1 LRR-domain.** Panel (**A**) 12 % (w/v) non-denatured PAGE of; lane 1 Benchmark^TM^ prestained protein ladder; lane 2, purified GmRLK18-1-LRR. Refolded GmRLK18-1-LRR showed presence of monomer as well as homodimer. Loading on SDS PAGE under reduced conditions showed only a single band [6]. Panel (**B**); A 12 % (w/v) non-denatured PAGE of; lane 1 Benchmark^TM^ prestained protein ladder; lane 2, GmRLK18-1 proteins detected by the anti-GmRLK18-1 antibody. Proteins were isolated from roots and refolded GmRLK18-1 showed presence of monomers as well as complexes in the correct position to be homo-dimers.

The LRR domains of GmRLK18-1 and GmRLK08-1 were expressed in *E. coli* by the same methods. Native page gels consistently showed 2 bands of 38.4 Kd and 76.8Kd for GmRLK18-1 (Figure 2A) but only a single bands at 38.1 Kd for GmRLK08-1 (data not shown). The 76.8 Kd bands could be eluted and electrophoresed on SDS-PAGE where the apparent size was halved to 38.4 Kd by denaturation. Proteins extracted from plant roots and subject to non-denaturing PAGE and Western transfer also appeared capable of maintaining homo-dimers and/or forming a hetero-dimers with another protein of similar mass and charge (Figure 2B). The predicted monomer band at 92.4 Kd electrophoresed more slowly than expected relative to the globular marker proteins as did the predicted homo-dimer 184.8 Kd band. Non-denaturing electrophoresis of proteins provides inaccurate estimates of size based on size and charge density. In this case the very low abundance RLK was a refolded protein following solubilization from the membrane bound fraction. Possibly the exposed trans-membrane domain or other unstructured elements reduced the rate of the proteins migration.

### Cross-linking between homodimers in GmRLK18-1 but not GmRLK08-1

From cross-linking with MTBS it was found that within 11 A^o^ from C57 to adjacent amino acids two sets of ion signals were observed which may both be assigned to the same region of a homodimer. The two biotinylated, trypsin digest derived, peptides that resulted from cross-linking started from residue 136 and ended with residue 169 (high abundance signal, mass 3791.97, 1 missed cleavage) and started with residue 131 and ended with residue 169 (low abundance signal, mass 4413.27, 2 missed cleavages). This means that C57 in the LRR or C215 in the whole protein are adjacent (>11A) to the region containing the H274N polymorphism (H133N in the LRR fragment). Therefore, the monomers are predicted to overlay one another but be offset by 79–112 residues. It should be noted that C215 would be close to one of the two intrinsically unstructured regions predicted. Also note in this structure the 10 of the 15 negatively charged residues (D + E) are predicted to be paired with 10 of the 22 positively charged residues (R + K) which causes the pI of the homodimer formed from the LRR domain expressed in *E. coli* to be nearly neutral (data not shown) rather than the pI 9 measured for the monomer [8,28]. In contrast there was no cross-linking with Gm08-RLK1 or the protein free controls

### GmRLK18-1 contained intrinsically unstructured elements

**Figure 3:**
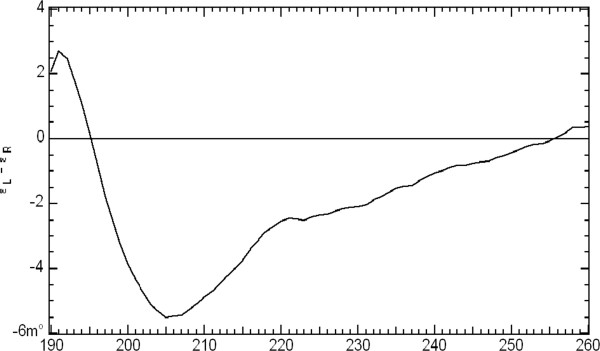
**Circular dichroism of GmRLK18-1-LRR in 20 mM phosphate buffer pH 6.0.** The CD data was processed using an integrated software package termed CDTOOL. The CD profile of GmRLK18-1 LRR is intermediary to LRRs with complete secondary structure (PGIP from *Phaseolus vulgaris*) and spectra generated from intrinsically unstructured proteins.

Circular dichroism spectroscopy of the refolded protein from the LRR domain was used to ensure the expressed proteins had refolded adequately. In fact the spectra showed most of the proteins were well folded but with spectra characteristic of interrupting and unstructured regions within the LRR domain(s) (Figure 3) [29]. CD showed a mixed secondary structure content (40% helix, 30% strand and turns). Interestingly, 21 percent of the LRR protein was predicted unordered or unstructured [6]. The unstructured regions intervene splitting the LRR domain in two. The intervening regions may underlie the difficulty in maintaining LRR solubility *in vitro* and alter the migration pattern during electrophoresis in non-denaturing gels.

### CLE peptide ligand binding by the LRR domain

The affinity of binding for two LRR domain proteins, GmRLK18-1 and GmRLK08-1 was measured with a set of 5 consensus motif peptides (Table 1) found among the CLE gene family expressed in soybean roots [30]. In addition, one CLE domain found in nematode secretions was tested. As a control the peptide used to raise the anti-RLK18 antibody was tested in the same assay. It contained an LRR repeat unique to the GmRLK18-1 class of proteins. There were 6 separate motifs among the eight GmCLE peptides as 3 of the motifs were present in short (12 residues) or long peptides (28–32 residues) in the plant secretome. The GmRLK18-1-LRR had highest affinity for short peptides in general (14–45 nM) and longer peptides were 2–3 fold less strongly bound. Peptides GmCLE34 (14 nM) and T (20 nM) were bound most strongly followed by CLV3 and its nematode ortholog N (29–30 nM). These binding constants were within physiological ranges and suggest the LRR domain can bind multiple peptide ligands. Each of the ligands was found *in vivo* as part of a signal cascade that alters plant development [25-27,29,31]. In contrast, to the set of peptides associated with developmental controls, the peptides involved in the control of nodule symbiosis GmRIC1 and GmNIC1 (2, 2L and 30) were bound weakly by the GmRLK18-1 derived LRR peptide.

**Table 1:** Sequences of CLE like peptides and control peptides used in binding assays

			**Kd (nM)**	
**Sequence**	**Name**	**Synonyms**	**RHG1**	**RHG4**
RLAPGGPDPQHN	2	GmNICI, LjCLE-R2 and d LjCLE-R1	45	96
DLPLAPADRLAGGPDPQHNVRAPPRKP	2L	GmNICI, LjCLE-R2 and d LjCLE-R1	142	338
RLAPEGPDPHHN	30	GmCLE30, GmRICI	44	84
AHEVPSGPNPISNR	T	GmTDIF, ZeTDIF	20	204
SKRRVPNGPDPIHNR	36	GmCLE34, AtCLE36, MtCLE36	14	52
RAELDFNYMSKRRVPNGPDPIHNRRAGNSGR	36L	GmCLE34, AtCLE36, MtCLE36	49	51
RTVPSGPDPLHH	3	GmCLE3, AtCLE3, AtClv3 unmodified	29	50
KGLGLHEEELRTVPSGPDPLHHHVNPPRQPR	3L	GmCLE3, AtCLE3, AtClv3 unmodified	65	142
KRLSPSGPDPHHH	N	HgCLV3	30	78
CTLSRLKTLDISNNALNGNLPATLSNLS	L	GmLRR, GmRLK18-1	36	135

Consensus sequences within the peptides were underlined. Dashed underline was the LRR peptide fragment used to estimate the Kd of dimerization and to raise the anti-Gm18RLK-1 antibody. Complete annotations can be found in [30]. Binding constants were calculated from titration experiments.

The GmRLK08-1 LRR domain (from the RLK protein at *Rhg4*) showed a lower affinity for most of the CLE peptides tested (50–338 nM). However, the long and short versions of GmCLE34 and short version of GmCLV3 bound with the highest affinity (50–52 nM) suggesting these were among the ligand signals integrated by GmRLK08-1. The nematode peptide HgCLV3 was bound weakly (78 nM). This result would agree with the conclusion that GmRLK08-1 protein was not the sole element underlying the resistance reaction encoded at the *Rhg4* locus [9]. The GmRLK08-1 LRR domain protein bound very weakly to the symbiosis associated GmRIC and GmNIC, as did GmRLK18-1. Unlike GmRLK18-1 the GmRLK08-1 protein bound weakly to GmTDIF. Therefore, the LRRs showed distinct peptide ligand specificities reflecting their different sequence and structures.

Estimates of the Kd for LRR domain dimerization could be made from the peptide L which contained one LRR motif. The apparent Kd for dimerization of 36 nM for this region would suggest the whole domain homo-dimerization constant be less than that. *In vitro* both proteins extracted from roots and LRR domain peptides solubilized from *E. coli* showed evidence that about half the proteins existed as monomers and about half as homo-dimers (Figure 2). This equilibrium is maintained across a wide range of concentrations of protein and salt. It will be of interest in future to see if ligand binding can alter this equilibrium.

### Whole protein ligand binding by the LRR in Far Western analyses

**Figure 4:**
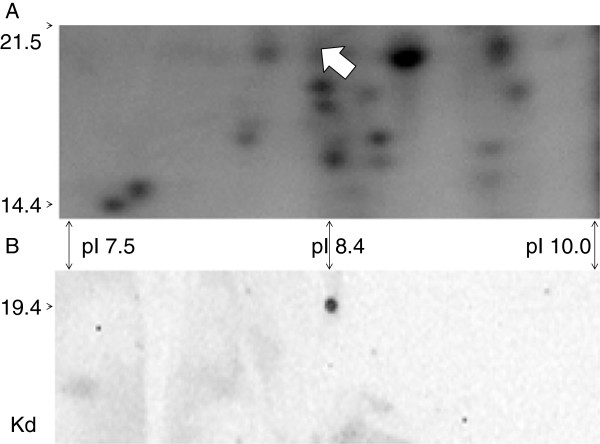
**Far-Western analysis of soybean root proteins at 24 dap (10 dai) probed with the LRR domain of GmRLK18-1.** Panel (**A**): Shown is a portion of a 2D gel (14.4-21.5 KDa; 7.5-10.0 pI) from 34–23 (resistant) SCN inoculated total root proteins with spots visualized with silver staining. Panel (**B**): Proteins transferred to a membrane and probed with purified GmRLK18-1 LRR domain and 6X his-RHG1. Anti-His-HRP was used as the secondary probe. The single spot identified (arrowed) was excised from the duplicate gel and analyzed by Q-TOF (MS-MS) to identify a cyclophilin as a GmRLK18-1 LRR domain interacting partner.

Far Western analyses of total root proteins separated on 2 D gels showed a single interacting partner was detected that was different at 10 and 42 dai with SCN (Figure 4). At 10 dai (24 dap) the feeding site has just developed and the resistance reaction has begun. The only protein avidly bound to the LRR at this stage was a cyclophilin, with 24% identical peptide matches to 2 regions of gi 17981611 (gb AAL51087.1), with Score of 284 and Expect value of 1e^-75^. The cyclophilin protein was 182 amino acids long and was found on the gel as expected from DNA derived amino-acid sequence prediction at 19,392 d and pI 8.38 (Figure 4). The abundance of the protein spot did not change either in response to SCN inoculation, *F. virguliforme* inoculation or plant genotype here or in earlier studies [31].

**Figure 5:**
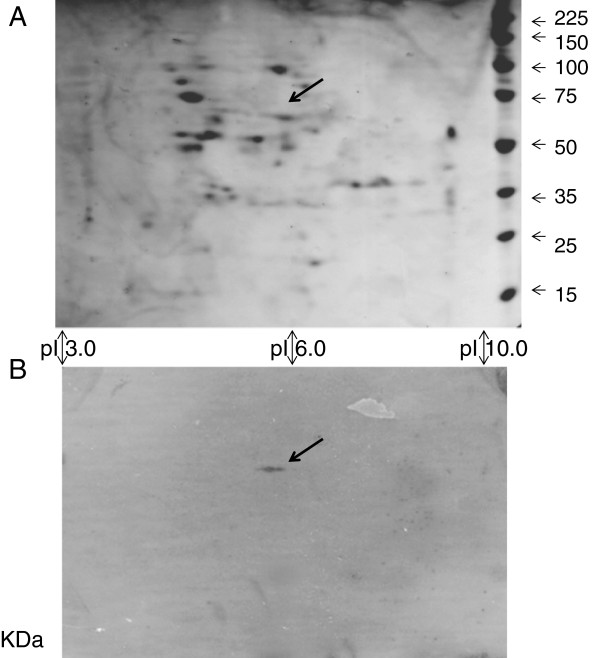
**Far-Western analysis of soybean root proteins at 42 dap (28 dai) probed with the LRR domain of GmRLK18-1.** Panel (**A**): Shown is a whole 2D gel (6.5-116.0 KDa; 3.0-10.0 pI) from 34–23 (resistant) SCN inoculated total root proteins with spots visualized with silver staining. Panel (**B**): Proteins transferred to a membrane and probed with purified GmRLK18-1 LRR domain and 6X his-RHG1. Anti-His-HRP was used as the secondary probe. The single spot identified (arrowed) was excised from the duplicate gel and analyzed by Q-TOF (MS-MS) to identify methionine synthase (GI: 33325957) at 84.2 KDa and pI 5.93 as a GmRLK18-1 LRR domain interacting partner. The other 3 proteins were of higher abundance and so not likely to be specific interactions.

By 28 dai (42 dap) cysts were mature (susceptible lines) or mostly dead (resistant lines). The protein binding most strongly to the LRR domain was a secreted methionine synthase from soybean. The methionine synthase protein (gi: 33325957) at 84.2 KDa and pI 5.93 was the strongest interacting partner (Figure 5). The abundance of the protein spot did not change either in response to SCN inoculation, *F. virguliforme* inoculation or plant genotype here or at 10 dai [31].

Proteins that contained CLE motifs were not detected at either time point (10 or 42 dai). The CLV3 like proteins in soybean range in size from 3,329-15,332 d and pI 5.4-11.9 but the active peptide ligands are much smaller (12–30 amino-acids), not abundant and so would not be present on the 2D gels.

## Discussion

Shown here were the structures and abundances of proteins that interact with the LRR of GmRLK18-1[28,31]. In addition distances between homodimer subunits were mapped and measured with established techniques [32,33]. These experimental data will be compared with models [34-42] based on homologies in this Discussion.

### Structure and function

The LRR domain of GmRLK18-1 was shown to tend towards homo-dimerization or ligand binding with equal avidity. Unstructured regions were detected within it. More than 30 percent of human proteins have unstructured regions within them [42]. Unstructured proteins offer advantages over globular/fixed proteins by potentiating low affinity transient interactions with a number of targets (lack of structure allows degeneracy) [43]. It is likely that the binding of interacting partners or pathogen effectors dictate RHG LRR domain structure. Many examples in cell cycle regulation, transcriptional regulation and cell signaling involve unstructured proteins. These include BRI1 [29,31] zinc-fingers, the ACTR cofactor in tumor development and the P53 involved in cell-cycle control [44]. Unlike structured proteins, most unstructured proteins have low affinities for their partners [42,45-47]. This may provide plasticity in cells needing a swift response to external or internal stimuli.

### Peptide ligand binding and phenotypic effects

CLE-like protein derived consensus peptides are defined set of peptides found in plant genomes and involved in both short and long distance signaling [26,30,48-51]. Five of the 16 consensus motifs found among CLE peptides are expressed in roots (Table1), as was the GmRLK18-1. The GmRLK18-1 LRR domain had a strong binding constant for GmCLV3 and N that are thought to be involved in meristem differentiation [25]. During SCN pathogenesis a new meristem is initiated to bring a tracheary element close to the feeding site, N might mediate that and be detected by the resistance protein GmRLK18-1. T is the tracheary element differentiation inhibitory factor (GmTDIF) that might provide inhibition of feeding site induced developmental processes during defence. GmCLE34 peptides were produced in pro-vascular tissues [48]. CLE domains thought to be involved in symbiosis [26] were not strongly bound suggesting they were not ligands of physiological relevance, although nematode parasitism does decrease nodulation [8].

In order to determine whether CLE peptide binding would have an effect in planta twelve plants were used for a root dip assay. SCN susceptible plants that had been infested with HgType 0 (isolate JB3) were depotted at 10 dai and a 0.5g root sample taken. Roots were then dipped in CLE peptide TGIF or HgCLE in water at 50 pM concentration and returned to the infested soil. Immediate wilt was observed among the plants dipped in CLE peptides but not the water controls (Additional file 1: Figure S1). Wilting and stem bending was more severe in X5 (panel A-D) plants that Westag 97 (panel E-H) plants. Within one hour of dipping in CLE peptide stem bending was observed (panel B and F) and maintained, though compensated for with an S bend in X5 plants, until roots were harvested at 28 dai (panel D). The roots were harvested 18 days after CLE peptide treatments and the number of cyst counted (Table 2). The plants dipped in CLE peptides showed significantly lower numbers of SCN females suggesting a resistance reaction had been induced.

**Table 2:** Association of CLE treatments with resistance to SCN JB3 and mean root growth in non-transgenic lines

		**Root**		**Root mass**		**SCN**
Line::gene	SCN infested	mass (g)		Range (g)	n	FI (%)
X5	No	1.05		0.81-1.44	15	0±0.0
X5	Yes	0.98		0.73-1.31	15	100±13
X5 + HgCLE	Yes	1.42		0.95-1.81	5	15+6
X5 + TDIF	Yes	1.40		0.92-1.78	5	8+3
Westag97	Yes	4.2		3.5-4.8	5	120±13
Westag97 + HgCLE	Yes	3.14		2.66-3.53	4	10+3
Westag97 + TDIF	Yes	3.10		2.65-3.51	4	5+3

SCN female index in greenhouse grown seedlings at 28 days after SCN infestations. Pots were watered daily with 100 ml. Female index (FI) was the mean percentage of cysts of Hg Type 0 found on five plants per repetition compared to a susceptible genotype Essex. Plant treated with CLE peptides received 50 pM dip treatments with HgCLE or GmTDIF.

### Protein ligand binding

The cyclophilin detected (Figure 4) was one of thirty encoded in the soybean genome suggesting it was a specific RHG1/RFS2 interacting partner. A role in pathogenesis for cyclophilins would be in agreement with [51]. The roles of cyclophilins include small molecule binding and receptor interactions [52]. The cyclophilin may induce a structural change in the LRR-domain probably by peptidyl-prolyl cis-trans isomerase activity (confirmation by NMR will be attempted in future experiments).

In a second Far Western analysis using older roots (42 day) later in the infection process (28 day) an S-adenosyl-L-methionine synthase was detected (Figure 5). Methionine synthase is increased in abundance and found in the secretome during fungal pathogenesis of plants [53]. Methionine synthase has a well-defined role in defense as a provider of a supply of methyl units. Recent experiments have shown that during infection of diploid wheat (*Triticum monococcum*) by the fungus *Blumaria graminis* f. sp. *tritici* there was a rapid synthesis of 12 proteins that are involved in the pathways of biosynthesis and supply of methyl units to lignol biosynthesis [54]. Methionine synthase was one of the genes shown to be highly induced at an early phase of infection in the epidermis. The expression was linked to host cell wall apposition formation and suggested that the pathways for synthesis of methyl units are transcriptionally activated and that this activation was for the host defense response. Cell wall appositions form during the late stages of the SCN resistance reaction in *G. max*[4-10]. Another possible role for methylation is that arginines in the LRR may be methylated during pathogen responses. Alternately it may be indirectly linked to developmental control during pathogenesis by altering the site and amount of ethylene production [52].

### GmRLK18-1 models

To understand structure-function details for GmRLK18-1, a three dimensional structural model was constructed. The generated model employed homology as well as *ab initio* predictions. The model endeavors were difficult as the most suitable tertiary structure homolog had low homology to GmRLK18-1. Consequently *ab initio* modeling was used in the final structural prediction. PRI [23,39] was used as the template structure (2bnh) from among the top hits generated by the 3D jury server. PRI was a 450 residue leucine rich repeat protein with a molecular weight of 49 kDa. The protein interacts and forms tight complexes with certain ribonucleases. Structure prediction was based on algorithms which select near-native conformations on the basis of discriminatory scoring functions. The models were first generated at 4–6 Å RMSD using the amino terminus domain of GmRLK18-1 (amino acids 52–440), which encompassed the LRR structural motif. In SCOP database the LRR domain (of both plants and all LRRs) was divided into 3 super families; (1) PRI-like (regular structure consisting the beta-alpha repeat); (2) L-domain like (less regular); and (3) both outer-arm dynein light chain-like and PGIP-like (beta-beta-alpha superhelix). GmRLK18-1 was predicted to be both PRI-like and PGIP-like. Several knowledge-based scoring functions have been developed with varying degrees of success [40,41]. These functions usually compile statistics from databases that contain experimentally determined structures, and use such statistics to test the probability of a given conformation to be native-like. Proteins of intermediate structure between two super-families often confound the programs.

### Model validation

ProcheckTM analysis of the LRR domain of GmRLK18-1 showed that most stereochemical parameters fell within accepted values for structures with resolution of 2.3 Å (Additional file 2: Table S1). As a resolution value cannot be assigned to the predicted structure of GmRLK18-1, the values are for comparison purposes only. Ramachandran plots for the model showed approximately 71 percent of residues in most favored regions and 26 percent of residues in allowed regions. Less than 1 percent (3) of the residues were in disallowed regions on the Ramachandran plot (Additional file 3: Figure S2, Additional file 4: Table S2).

The PROCHECK analyses of the crystal structure of PRI (Additional file 3: Figure S3) showed approximately 80 percent of the residues were in the most favorable regions and 20 percent in additionally favorable regions. Therefore, about 10% of most favored residues present in the template were lost during GmRLK18-1 model generation.

**Figure 6:**
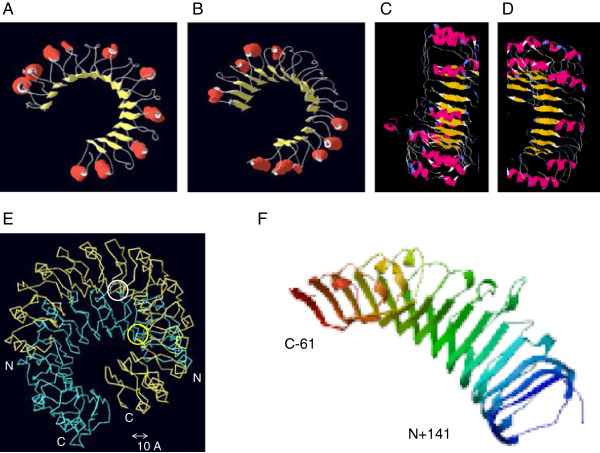
**Predicted structures of the GmRLK18-1 LRR monomer.** Panels (**A-D**) The β sheet regions are shown in yellow and helical regions in red. The modeling results suggest LRRs of GmRLK18-1 at *Rhg1/Rfs2* (panel **A**) and GmRLK8-1 at *Rhg4* adopt horse shoe type architectures. In GmRLK18-1, the N and C terminal helices are longer and the shorter helices are evenly spaced in the repeats. Also seen are 4 pockets in the protein where helices do not form and the unstructured regions are the two nearest the C terminus. In GmRLK18-1 prediction, the N and C terminal helices are longer and the shorter helices are evenly spaced in the repeats whereas in the GmRLK8-1 prediction, the helices are unevenly spaced and are present only at the N or C terminal. Panel **C** shows the predicted GmRLK18-1 structure looking at the concave surface and panel **D** was looking at the convex surface. Panel (**E**) GmRLK18-1 was modeled as a crystal homo-dimer based on the RI template. The homo-dimer interface was held together by anti parallel β sheets involving many residues from each monomeric chain. Chains were offset by about 90 residues. Circled in white is the cysteine less than 11 A from the partnering homo-dimer chain as detected by cross-linking, circled in yellow is the cysteine not near the dimer interface. (**E**) The predicted structure by SWISS-PROT [49] for only the LRR domain from amino acid 141–435 of GmRLK18-1 that was expressed in *E. coli*. The N-terminus lacked the signaling peptide. The C terminus was 61 amino acids (−61) short of the start of the trans-membrane domain.

The model suggested that the LRR domain of GmRLK18-1 (Figure 6) adopts a horse-shoe type architecture similar to the crystal structure of PRI and unlike the solenoid structure of monomeric BRI1 [18,19]. In both the template and modeled protein, the long ß sheets are parallel to the helices present on the inner circumference of the proteins. In GmRLK18-1, the helices and sheets were joined by loops. Further, the N and C terminal helices were longer and the shorter helices were evenly spaced in the repeats (Figure 6).

The lack of motif conservation in known LRR domains [2] suggested that LRRs involved in pathogen recognition have a greater need to adapt to the constantly changing pathogen population. Some structural elements mainly helices, present in the template PRI structure [39] were missing in the GmRLK18-1-LRR protein (Figure 6). Surprisingly the loss of helical contact seems to be at odd numbered helical turns in the solenoid protein (helix numbers 5, 7, 9 11 and 13).

### GmRLK18-1 LRR modeled as a crystal homo-dimer

Native PAGE of GmRLK18-1 showed a monomer as well as a homo-dimer band (Figure 2A and 2B). Therefore, the GmRLK18-1-LRR protein was modeled as a crystal homo-dimer (Figure 6). This modeling was based on either the crystal dimer from decorin template [21] or an amino-peptidase or the RI dimer complexed with angiogenin (1a4y) [23]. The model developed based on the RI dimer agreed with the experimental evidence from MTBS cross-linking and was shown in Figure 6. The models based on decorin and the amino-peptidase did not agree with experimental data and was rejected. Briefly, from cross-linking from C57 two biotinylated, trypsin digest derived, two peptides from one region of a homo-dimer resulted from cross-linking. This means that C57 in the LRR or C215 in the whole protein were adjacent (<11A) to the region containing the H274N polymorphism (H133N in the LRR fragment). Therefore, the monomers are predicted to overlay one another but be offset by 79–112 residues. It should be noted that C215 would be close to one of the two intrinsically unstructured regions predicted. Also note in this structure the 10 of the 15 negatively charged residues (D + E) are predicted to be paired with 10 of the 22 positively charged residues (R + K). That would cause the pI of the homodimer formed from the LRR domain expressed in *E. coli* to be nearly neutral. Neutral proteins have been detected in non-denaturing pI measurements using the DIABLA assay systems (data not shown) [28] rather than the pI 9 measured for the monomer (Figure 2) [8].

XA21, one of the closest homologs of the GmRLK18-1-LRR was believed to function via homo-dimer formation [11,24]. Other LRRs implicated in development have also been reported to homo-dimerize. For instance, an *in vivo* study of the *Arabidopsis* Somatic Embryogenesis Receptor Kinase (SERK) showed evidence of a SERK homo-dimer whilst CLV1 LRR domain heterodimerizes with CLV2 LRR domain (25].

To evaluate stability of the mutant structures, the pseudo energy was calculated using RAPDF. The RAPDF scores suggest that all 3 mutations may affect the stability of the dimeric protein (Additional file 5: Table S3), though 2 these 3 residues were absent from the LRR fragment expressed in *E. coli*.

### Halplotype and allotype analysis of GmRLK18-1

Previously a perfect association between allele 1 (Forrest based resistance) and resistance to the SCN (Hg Type 0 or race 3) was shown in three segregating populations and an association study of unrelated PIs [5]. Recently, previously susceptible plants transgenic with the Forrest allele of GmRLK18-1 were found to be resistant to both SCN and SDS [8]. Based on multiple sequence alignment of predicted GmRLK18-1 haplotypes, a quantitative trait nucleotide in the LRR of *rhg1* was inferred, that alters A87 to V87 in the context of Q115 and H274 rather than K115 and N274 (Additional file 6: Figure S3). There is a closely linked copy number variation that may also contribute to resistance [8].

Effects of the non synonymous point mutations on the RLK extracellular domain protein monomer stability was analyzed using the Fold X algorithm (http://fold-x.embl-heidelberg.de) [46]. Of the 3 mutations that could discriminate between resistant and susceptible *rhg1* alleles, the A to V substitution at amino acid 87 was predicted to make the most significant change to the free energy of folding (Additional file 7: Table S4). The changes Q to K at position 115 and H to N at position 274 have an opposite effect on protein stability. However, the predicted absolute folding free energy values are not usually accurate compared to proteins with determined structures. Hence the absolute values were of little significance and only the values obtained from free energy differences between the 3 mutants and the wild type structure were used for relative comparisons. Functionally important residues in the GmRLK18-1-LRR were also distinguished from structural residues using a recently developed algorithm [47]. Each residue in the GmRLK18-1 LRR protein was analyzed on the basis of (a) a sequence conservation score based on multiple sequence protein alignment and (b) the free energy difference between the naturally occurring residue and the potentially optimal residue at that position. The analysis showed that the alanine residue at position 87 was the optimal residue in addition to the naturally occurring residue in the Peking cultivar. Both these results suggest that alanine 87 may be involved in providing stability to the protein. The remaining 2 amino acid substitutions may have an effect on the stability of the protein dimer.

## Conclusions

This report describes a functional analysis and structural prediction of the LRR domain from a RLK protein involved in plant pest and disease resistance. Structural predictions were made and validated. The results showed that the isolated LRR of GmRLK18-1 binds peptides found in both nematode and plant secretions with high affinity. A question might arise over whether isolated LRRs behave in the same way as in whole RLKs and RPKs. However, the LRR was previously shown to form an active structure by CD [27]. Homo-dimerization was detected in 3 different assays (2 *in vitro*) and the avidity estimated by a peptide binding assay. Larger proteins were also shown to bind the LRR domain including cyclophilin, methionine synthase and a protein of the same mass and charge as the RLK monomer. Therefore, the isolate LRR behaved in ways similar to the RLK. The LRR domain was large and appeared to be binding different ligands. The RLK might integrate those signals into a single appropriate response.

The predicted structure of the RLK contrasted with the template PRI or monomeric BRI1 in a number of ways. First, the predicted model lacks some of the successive alpha helical regions present in PRI and BRI1. This may account for the unstructured content that was determined by circular dichroism spectroscopy. Secondly, it was predicted that the N terminal helical repeats were longer whereas the internal helical regions were evenly placed throughout the protein. In most other aspects, the model was very similar to the PRI template. Future predictions may be based on plant RLK homo-dimers of similar lengths if any structures become available or if the GmRLK18-1 homo-dimer can be crystallized. In the absence of that the prediction employed here can be used for extensive structure refinement by further cross-linking experiments.

Some important features of the candidate GmRLK18-1 protein were predicted and validated. The three polymorphisms to the C-terminal side, A87V, Q115K, and H274N may affect protein stability. The quantitative trait nucleotide underlying H274N that differentiates the Peking allotype (for resistance) from all others may have structural or functional significance. That amino acid polymorphism, H274N, may play an important role in the stability of the monomer as well as the homo-dimer. Future experiments will involve mutation of this amino acid and correlating it with GmRLK18-1 stability and/or function in binding the CLE like peptide motifs found in all plants and their many of their pathogens [30].

## Methods

### Plant materials

Cultivars ‘Essex’ and ‘Forrest’, derived near isogenic lines ‘EF34-3’ and ‘EF34-33’, ‘X5’ and ‘X5 transgenic with the Gm18-1 RLK’ (X5::RLK) were grown in a growth chamber at 26 C as described previously [6,7,31].

### SCN inoculations

Soybean plants were grown in 5 l buckets, each containing 20 cones in a randomized block. Each bucket contained a 1:1 ratio of sand soil mix. The containers were placed in a water bath set at 26°C in the SIUC greenhouse. Growth conditions were a 14h light cycle, aerial day time temperature of 30°C and a nighttime temperature of 22°C. Infections were with an Hg Type 0 SCN population (JB3). Inoculated were 2,000 eggs to each 14 day old seedling. Inoculated soybean plants were removed from the cones; at 10 dai (24 dap) and roots sample of 0.5 g frozen or at 28 dai (42 dap) and roots frozen after cyst numbers counted. Experiments were repeated. Some experiments used a growth chamber for assays of SCN and root growth. The conditions varied from the greenhouse as follows. The whole chamber was set at 26°C. The humidity was maintained at approximately 40-50% judged by indicator cards.

### GmRLK18-1 and GmRLK08-1 LRR domain expression in *E. coli*

The GmRLK18-1-LRR was expressed and purified from pET30a in *E. coli* as reported previously [6]. The RHG4-LRR was cloned and expressed by the same methods during this work in pET28a. Briefly, the LRR residues from 141–435 (Figure 1) were expressed in *E. coli* strain BL21 from an IPTG inducible promoter at 10 C. The LRR proteins were isolated by precipitation of inclusion bodies and washing with extraction buffer. Pure inclusion bodies were partly solubilized by incubation in 2M urea and 10% (w/v) glycerol. Folded proteins were selectively solubilized by this low concentration of urea. Proteins were further purified using immobilized metal ion affinity chromatography (IMAC) with Ni-NTA agarose. Proteins were aliquoted and stored at −20 or −80 C until use.

### Circular dichroism spectroscopy and determination of unstructured regions

CD spectroscopy of purified GmRLK18-1 was carried out as reported previously [6]. Briefly, to remove the urea proteins were dialyzed against 0.5M urea, 5mM reduced and 2mM oxidized glutathione and 2.0M arginine. The protein was further dialyzed against 1.0M arginine, glutathione couple before dialysis against, pH 6.0, buffered sodium phosphate. Proteins had to be used within 48 h to avoid precipitation once they were urea free.

CD spectra were measured in a quartz 0.5mm path length cuvette at 25°C for protein concentrations of 0.2–0.3mg/ml. An Aviv 62-DS spectrometer (Lakewood, NJ) was used for the analysis of native, partially folded and unfolded proteins. The protein far-UV spectra were recorded over a wavelength range of 190–250nm at an averaging time of 1 s and 3 scans averaged over the far UV wavelength range. The CD data was processed using an integrated software package termed CDTOOL. Secondary structure content was determined with CDSSTR at the dichroweb server (http://dichroweb.cryst.bbk.ac.uk/html/home.shtml). The Prelink^TM^ prediction algorithm [29] was used to probe the LRR domain for potential unstructured regions.

### Native PAGE to detect monomers and dimers

The 12% (w/v) native PAGE of GmRLK18-1 was performed using protein extracts from soybean roots [31] or *E. coli*[6]. Bands observed on native PAGE that were inferred to be the GmRLK18-1 LRR domain monomer and homo-dimer from *E. coli* were eluted. The eluants were again electrophoresed under non-denaturing and denaturing conditions on 12% (w/v) SDS PAGE using established protocols [6]. Protein pIs were measured after [8] and [28].

### LRR domain modification protocol with the MAB reagent

The 2M urea was removed from the solubilized LRR using the Sephadex G-25 size-exclusion column chromatography. The resin was initially equilibrated with an equilibration buffer (21mM Tris-Cl pH 8.0, 1mM EDTA, 0.01% (v/v) NP-40, 5% (v/v) glycerol and 200mM NaCl) in a ratio of 1g/10ml of resin to equilibration buffer and packed into a 1ml BD™ syringe tubes by spinning the tubes for 5 minutes at 1,000 *g* and 4°C. Thereafter, the protein was applied onto the packed column and centrifuged again for 5 minutes at 1,000 *g* and 4°C. A ten-fold excess of 2-[N2-(4-azido-2,3,5,6-tetrafluorobenzoyl)-N6-(6-biotinamidocaproyl)-L-lysinyl]ethyl methanethiosulfonate, (MAB; Pierce Biotechnology, Rockford, IL) reagent dissolved in dimethyl formamide was used to modify 15 μg (3.5 pM) of the LRR in the dark for 1hr at room temperature after [32]. The MAB bound to the free cysteine at the N terminus of the LRR during this step. Later, the excess reagent was removed from the reactions via Sephadex™ G-50 size-exclusion column chromatography.

### UV cross-linking and label-transfer procedure

The MAB modified LRR domain proteins (3.5 pM) were allowed to freely bind and/or dissociate from their homo-dimer partners by equilibrating them at 30°C for 30 minutes in a 12.5 μl reaction containing 30mM NaOH-HEPES [pH 7.8], 60mM NaCl, 5mM MgCl_2_, 5% (v/v) glycerol and 0.1mg/ml BSA. UV-irradiation (Spectroline BIO-VISION UV/white light transilluminator, 310 nm and 2.65 mW/cm^2^ at a distance of 8 cm) for 2 min caused the biotin label to covalently link to the nearest residue (< 11.1 A away) [33]. The biotin label was transferred to that residue by adding DTT to a final concentration of 100mM to break the disulphide bond to the cysteine.

### Preparation of protein for mass spectrometry and enrichment of the biotinylated peptides

Initially, the LRR domains were digested with trypsin (dissolved in 1mM HCl), in a ratio of 1:5 of the enzyme: substrate, for 3 hours in 25mM ammonium bicarbonate at 37°C. Then the reaction was stopped with the protease inhibitor PMSF in (~20 fold molar excess to trypsin) before passing through a monomeric avidin column (with a binding capacity of biotinylated proteins to be ~1.2mg/ml) which was already blocked with blocking buffer (4mM d-biotin dissolved in 1x PBS) and also washed with 12 column volumes of elution buffer (0.4% (v/v) trifluoro-acetic acid and 40% (v/v) acetonitrile). The biotinylated peptides were eluted from the column using 5 column volumes of the elution buffer (200 μl). Samples were sent for peptide fragment size estimation to the MS facility at SIUC for MALDI analysis. Briefly, a 1μl drop of trypsinized protein was added to 1 μl of MALDI matrix (5 mg/ml alpha-cyano-4-hydroxycinnamic acid in 50% (v/v) acetonitrile) and dried on a stainless steel plate. A Bruker Daltonics Microflex™ (Billerica, MA) time of flight mass spectrometer was used to analyze the sample with a pulse nitrogen laser set at 337nm with a 20 kHz repetition rate and ions that resulted were observed in the positive ion mode as the sum of 500 individual mass spectra over an m/z range from 1–10 thousand.

### Western and Far Western analysis with LRR domain probes

SDS–PAGE of total plant proteins from Essex and Forrest followed by Western hybridization was carried out by methods described previously [6,7] with the following modifications. For the Western hybridizations, a custom made antibody generated against the peptide CTL SRL KTL DIS NNA LNG NLP ATL SNL S from the LRR domain of RHG1 was used (Alpha Diagnostics, San Antonio, Texas). As a secondary antibody, an anti rabbit IgG HRP was used (GE healthcare, Milwaukee, Wisconsin).

For Far-Westerns the LRR domain was used as a probe to filters transferred from 1 or 2 D gels. As the secondary probe, anti-his antibody conjugated to HRP (1–10,000 dilution; Invitrogen, Carlsbad CA) was used. Spots and bands were picked manually and the proteins in them digested with trypsin and identified by direct infusion MS/MS following the methods in [31].

### Ligand binding assays

For *in vitro* assays potential peptide ligands were labeled with fluorescein-5-isothiocyanate (FITC) in a molar ratio of 10:1 at room temperature for 60 mins (G-Biosciences, St Louis, MO). Labeled peptides were stored at −20 C in 10% (v/v) glycerol until use. For ligand binding assays peptides were diluted to 50nM/ml in 20 mM phosphate buffer (pH6.9) in a cuvette and the amount of polarized luminescence measured in a luminescence spectrometer scan from 500–600 nm with excitation energy of 494 nm and a detection wavelength 520 nm. The excitation slit was set to 4 nm and the emission slit was set at 4 nm with a scan speed of 100 nm/min. Ligands were added to the cuvette in excess (50 nM) Peptides were added in 5 nM aliquots and the increase in polarization measured as fluorescence units. Base-line binding to non-CLE and non-LRR peptides was subtracted from the polarization units. Experiments were repeated 3 occasions with 3 different protein preparations. Dissociation constants (Kds) were calculated from double reciprocal Scatchard plots [31].

*In planta* assays used twelve plants of each of cultivar X5 and Westag 97 in two separate experiments over a month for a root dip assay [55]. SCN susceptible plants that had been infested with HgType 0 (isolate JB3) were depotted at 10 dai and a 0.5g root sample taken. Roots were then dipped in CLE peptide TGIF or HgCLE in water at 50 pM concentration and returned to the infested soil. Photographs were taken at 2 min, 5 min and 18 days after treatments.

### Bioinformatic analysis of GmRLK18-1

The nucleotide sequence for GmRLK18-1, the RLK encoded within the *rhg1* locus, was translated to its polypeptide sequence *in-silico* using the EXPASY translation tool (http://www.expasy.ch/tools/dna.html). The complete (855 amino acid) sequence obtained for GmRLK18-1 was analyzed for domain architecture (smart.embl-heidelberg.de). The LRR-domain (amino acid 52 to amino acid 440) was used for generation of models. The Apache server found at (supfam.mrc-lmb.cam.ac.uk/SUPERFAMILY) was used to predict the superfamily, fold and class of the protein. The ratio of synonymous to non-synonymous substitutions was analyzed by the web based algorithm SNAP (http://www.hiv.lanl.gov/content/sequence/SNAP/SNAP.html).

### Comparative and homology modeling of structures

The first model of GmRLK18-1 structure was generated using the RAMP software suite of programs (http://compbio.washington.edu; http://protinfo.compbio.washington.edu). The initial template and the corresponding sequence alignments of GmRLK18-1 were chosen from the 3D-Jury server (http://BioInfo.PL). The best template was decided by inspection of the consensus sequence of the GmRLK18-1-LRR protein and all template sequences (Additional file 8: Figure S4). Finally, these alignments were adjusted manually to obtain the best alignment for the LRR-domain. The crystal structure of the PRI (PDB entry 2BNH) was used as the template structure for modeling of GmRLK18-1.

The initial models were constructed with a minimum perturbation approach [34]. Variable side chains and main chains were constructed by using a graph-theory clique-finding approach, which explores a variety of possible conformations for the respective side chains and main chains and finds the optimal combination by using an all-atom scoring function. These approaches were described in detail previously [34-37]. Briefly, based on each individual alignment, initial models were generated by copying atomic coordinates for the main chain (excluding any insertions/loops) and for the side chains of residues that were identical in the target and template proteins. Models for residues that differed in side chain type were constructed using the SCWRL3 program. A set of possible conformations were generated for the main chain regions (loops) considered to vary in target with respect to the template structures. The models included potential insertions and deletions. Main chain sampling was performed using an exhaustive enumeration technique based on 14 discrete torsion angle states. Refinements of the *ab-initio* sampling protocols were also incorporated into the loop sampling technique [35,38]. All models were refined with ENCAD, and the best model was selected using the Residue all-atom conditional Probability Discriminatory Function (RAPDF).

The homo-dimer conformation of GmRLK18-1 was generated using the software package XtalView, according to the relative position of the homo-dimer conformation of a Ribonuclease inhibitor-angiogenin complex (1a4y). The structures of GmRLK18-1 allotypes (A87V, Q115K and H274N) were generated using SCWRL3. To evaluate the stability of the structures, their pseudo-energy was calculated using RAPDF.

### Structural model validation and analysis *in silico*

Protein structure validation checks, mainly the stereo-chemical quality of protein structure were performed using PROCHECK (http://www.ebi.ac.uk/thornton-srv/software/PROCHECK/). Analysis of main chain, side chain parameters and Ramachandran plot analysis used the PROCHECK algorithm. The 3 amino acid changes from the SCN Type I resistance allotype to the susceptible allotypes were analyzed further for functional and structural importance using two prediction servers (http://robetta.bakerlab.org/; http://fold-x.embl-heidelberg.de).

## Abbreviations

LRR: Leucine rich repeat; RLK: Receptor like kinase; CLE: Clavata like elicitor.

## Authors' contributions

DAL conceived of the study and all of the analysis. AS and JA carried out the Far Westerns. RS and JY carried out the homology modeling. SV developed the GmRLK08-1 expression vector. DAL and SK carried out the CLE binding assays. AG carried out the cross-linking experiment. DAL carried out the root dip assays. DAL drafted the manuscript. AS and JA provided critical review, interpretation of results and feedback. All authors read and approved the final manuscript.

## Supplementary Material

Additional file 1: Figure S1 Effect of exogenous treatments of roots of X5 and Westag 97 with CLE peptides a root dip assay. SCN susceptible plants that had been infested with HgType 0 (isolate JB3) were depotted at 10 dai (24 dag) and a 0.5g root sample taken. Roots were then dipped in CLE peptides TGIF or HgCLE in water at 50 pM concentration and returned to the infested soil. Wilting and stem bending was more severe in X5 (panel **B**-**D**) plants that Westag 97 (panel **F**-**H**) plants. Panels A and E were before depotting. Panels **B** and **F** were 2 min after dipping and repotting. Panels **C** and **G** were 15 minutes after treatment. Panel **D** and **G** were at root harvest 28 dai (42 dag). Cyst were abundant on the water treated plants but 10±6 were found on each of the 5 plants dipped in HgCLE or GmTDIF (Table 2). Click here for file

Additional file 2: Table S1 PROCHECK analysis of main chain parameters for the modeled GmRLK18-1 LRR structure. Stereochemical parameters such as percentage residues in allowed regions, omega angle standard deviation, the hydrogen bond standard deviation and the overall quality of the models are shown for the modeled GmRLK18-1-LRR and typical proteins resolved at 2.3 Å resolutions. Click here for file

Additional file 3: Figure S2 Ramachandran plot of homology-modeled structure of GmRLK18-1 based on porcine ribonuclease inhibitor template. Each amino acid residue is represented by a black dot. Red shows the most favored residue positions, yellow additionally allowed residue positions, beige residue generously allowed residue positions and white disallowed residue positions. Click here for file

Additional file 4: Table S2 Ramachandran plot statistics for GmRLK18-1 calculated from PROCHECK. Approximately 71 percent of the amino acid residues within the modeled protein were in allowed regions of the Ramachandran plot. An additional 26 percent of the amino acid residues were in additional allowed regions whereas 2.4 percent of the amino acids fell in generously allowed regions of the Ramachandran plot. Less than one percent residues (3) were in disallowed regions. Click here for file

Additional file 5: Table S3 RAPDF scores for 3 LRR mutants and the wild type GmRLK18-1-LRR. The RAPDF scores suggested that these mutations may affect the stability of the homodimeric protein although these residues were not directly implicated in the homodimer interface. Click here for file

Additional file 6: Figure S3 Sequence diversity among the seven GmRLK18-1 allotypes. Alanine at position 87 is only present in the ‘Peking’ sequence. Two additional changes Q to K at position 115 and H to N at position 274 are not exclusive to resistance type I. Click here for file

Additional file 7: Table S4 Effect of three non-synonymous substitutions on protein stability calculated from the Fold X algorithm (http://fold-x.embl-heidelberg.de). The computed free energy of folding and the change in free energy between the wild type protein (Peking allele) and the mutant proteins is shown. The H to N and Q to K change increase the free energy of folding, whereas the alanine to valine change results in a significant decrease in the free energy. (DOC 45 kb) Click here for file

Additional file 8: Figure S4 The 3D structures of LRR containing proteins showing high sequence homology to RHG1. Panel (**a**) shows the crystal structure of BRI1 (pdb id: 3RGX). Brassinosteroid recognition is mediated through the LRR domain of BRASSINOSTEROID-INSENSITIVE 1 (BRI1). BR1I exists as a monomer. The protein exists as a helical solenoid structure. (**b**) crystal structure of the Polygalacturonase inhibiting protein (PGIP). The PGIP protein (PDB id: 1ogq) is a cell wall localized protein that interacts with fungal endopolygalacturonases. (**c**) X-ray structure of the porcine ribonuclease inhibitor (PRI). The Leucine rich repeat of PRI (PDB id: 2BNH) forms tight complexes with ribonucleases thereby regulating RNA levels. PRI adopts a horseshoe configuration with the LRR motif composed of repeat beta-loop helix units. Click here for file
