# Supplementary figures and images for: Homo-dimerization and ligand binding by the leucine-rich repeat domain at RHG1/RFS2 underlying resistance to two soybean pathogens
Source: BMC Plant Biol. 2013 Mar 15;13:43. doi: 10.1186/1471-2229-13-43 (PMC3626623; doi:10.1186/1471-2229-13-43)

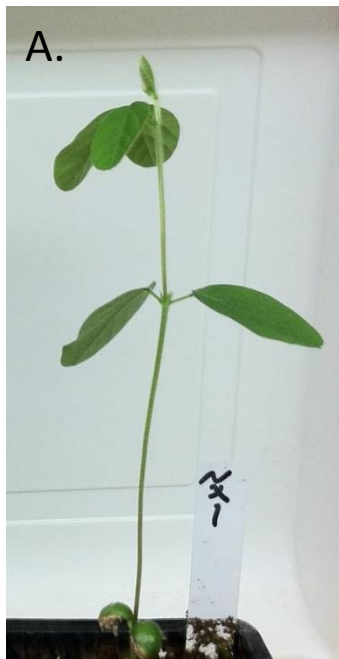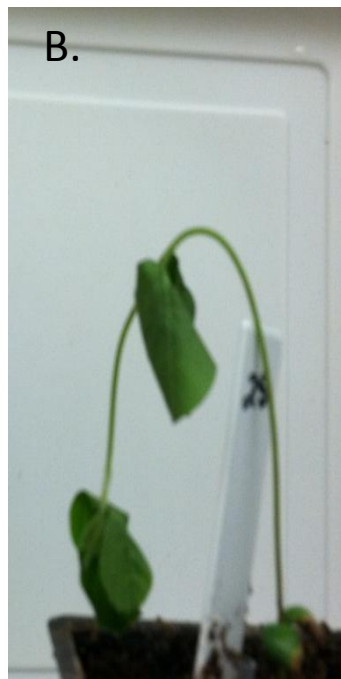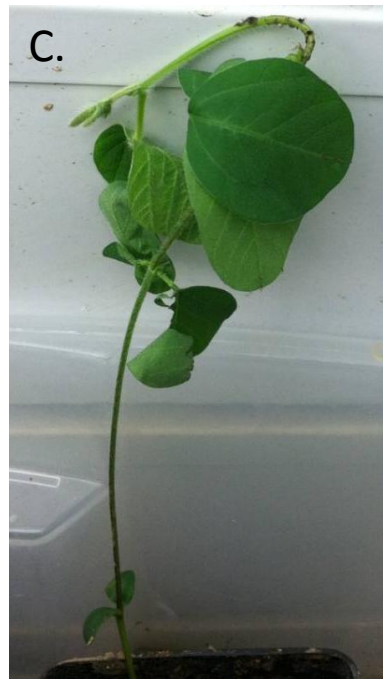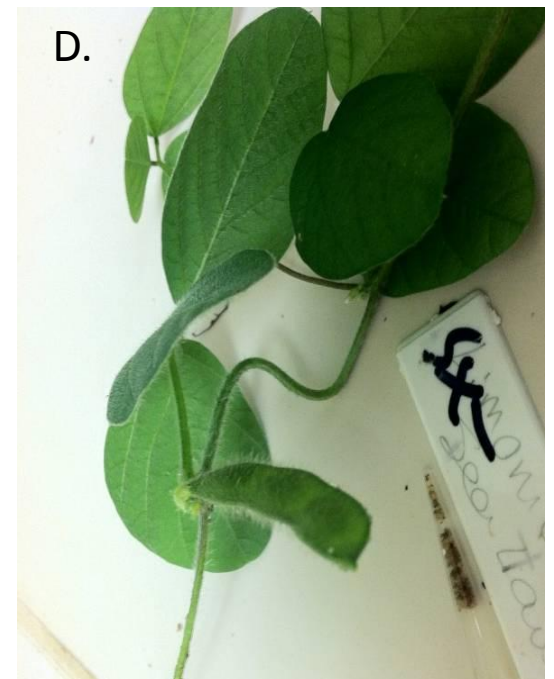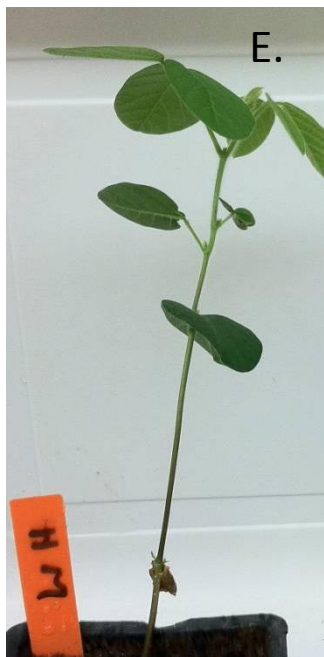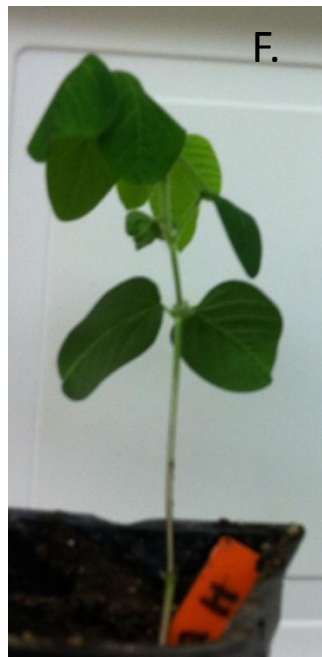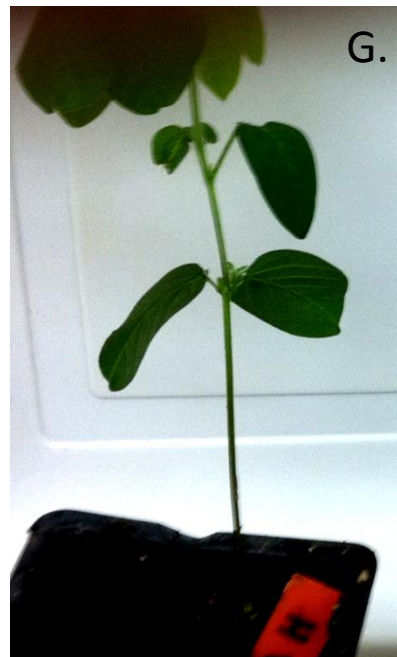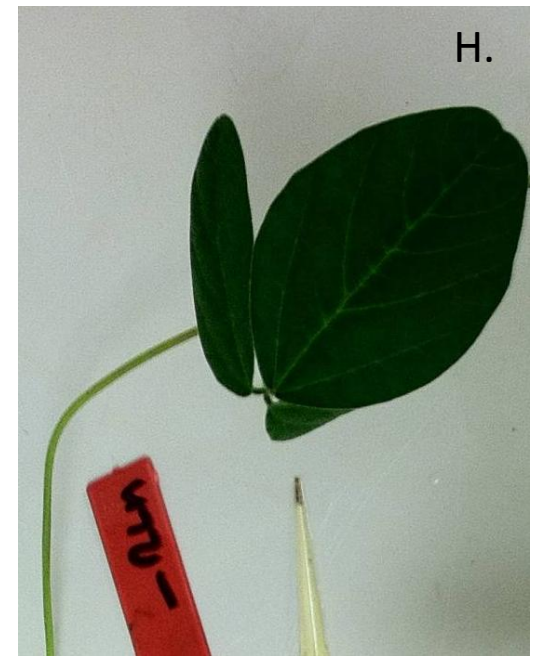

Supplement: Additional file 1: Figure S1 — Effect of exogenous treatments of roots of X5 and Westag 97 with CLE peptides a root dip assay. SCN susceptible plants that had been infested with HgType 0 (isolate JB3) were depotted at 10 dai (24 dag) and a 0.5g root sample taken. Roots were then dipped in CLE peptides TGIF or HgCLE in water at 50 pM concentration and returned to the infested soil. Wilting and stem bending was more severe in X5 (panel B-D) plants that Westag 97 (panel F-H) plants. Panels A and E were before depotting. Panels B and F were 2 min after dipping and repotting. Panels C and G were 15 minutes after treatment. Panel D and G were at root harvest 28 dai (42 dag). Cyst were abundant on the water treated plants but 10±6 were found on each of the 5 plants dipped in HgCLE or GmTDIF (Table 2). [file 1471-2229-13-43-S1.pdf]

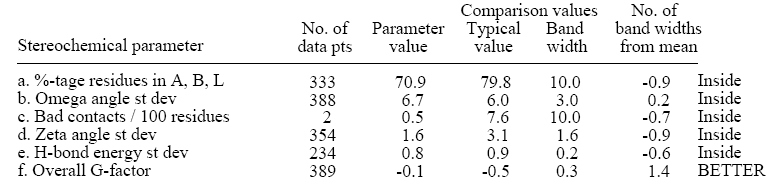

Supplement: Additional file 2: Table S1 — PROCHECK analysis of main chain parameters for the modeled GmRLK18-1 LRR structure. Stereochemical parameters such as percentage residues in allowed regions, omega angle standard deviation, the hydrogen bond standard deviation and the overall quality of the models are shown for the modeled GmRLK18-1-LRR and typical proteins resolved at 2.3 Å resolutions. [file 1471-2229-13-43-S2.doc]

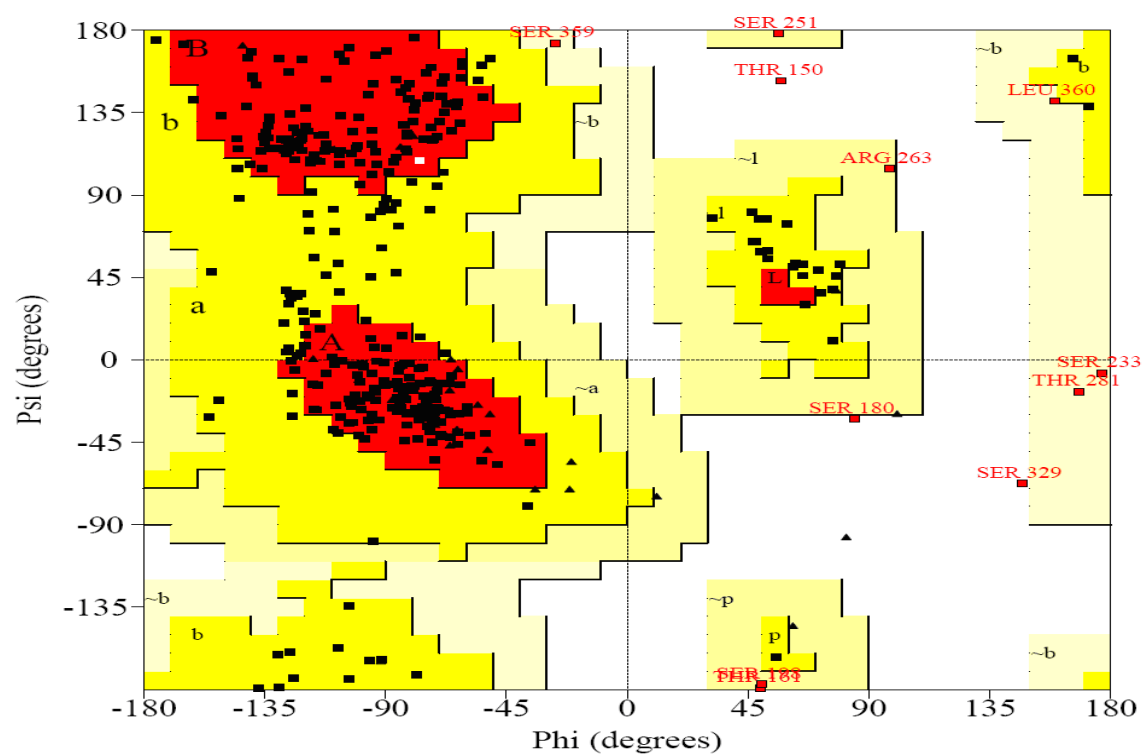

Supplement: Additional file 3: Figure S2 — Ramachandran plot of homology-modeled structure of GmRLK18-1 based on porcine ribonuclease inhibitor template. Each amino acid residue is represented by a black dot. Red shows the most favored residue positions, yellow additionally allowed residue positions, beige residue generously allowed residue positions and white disallowed residue positions. [file 1471-2229-13-43-S3.pdf]

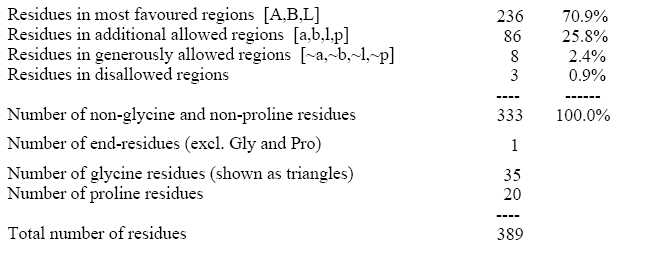

Supplement: Additional file 4: Table S2 — Ramachandran plot statistics for GmRLK18-1 calculated from PROCHECK. Approximately 71 percent of the amino acid residues within the modeled protein were in allowed regions of the Ramachandran plot. An additional 26 percent of the amino acid residues were in additional allowed regions whereas 2.4 percent of the amino acids fell in generously allowed regions of the Ramachandran plot. Less than one percent residues (3) were in disallowed regions. [file 1471-2229-13-43-S4.doc]
