# Supplementary material for: Homo-dimerization and ligand binding by the leucine-rich repeat domain at RHG1/RFS2 underlying resistance to two soybean pathogens
Source: BMC Plant Biol. 2013 Mar 15;13:43. doi: 10.1186/1471-2229-13-43 (PMC3626623; doi:10.1186/1471-2229-13-43)
Supplement: Additional file 5: Table S3 — RAPDF scores for 3 LRR mutants and the wild type GmRLK18-1-LRR. The RAPDF scores suggested that these mutations may affect the stability of the homodimeric protein although these residues were not directly implicated in the homodimer interface. [file 1471-2229-13-43-S5.doc]

GmRLK18-1 **mutants RAPDF Score**

GmRLK18-1_87A_V -40.63

GmRLK18-1_115Q_K -40.95

GmRLK18-1_274H_N -40.80

GmRLK18-1_WT -43.65
