# Supplementary material for: Homo-dimerization and ligand binding by the leucine-rich repeat domain at RHG1/RFS2 underlying resistance to two soybean pathogens
Source: BMC Plant Biol. 2013 Mar 15;13:43. doi: 10.1186/1471-2229-13-43 (PMC3626623; doi:10.1186/1471-2229-13-43)
Supplement: Additional file 6: Figure S3 — Sequence diversity among the seven GmRLK18-1 allotypes. Alanine at position 87 is only present in the ‘Peking’ sequence. Two additional changes Q to K at position 115 and H to N at position 274 are not exclusive to resistance type I. [file 1471-2229-13-43-S6.pdf]

Allele	Amino acid	R1	R2	R3	R4	S1	S2	S3	S4	S5
SNP0	71	V	V	V	A	V	V	V	V	V
SNP1	87	A	V	V	A	V	V	V	V	V
SNP2	115	Q	Q	Q	Q	K	K	K	Q	Q
SNP3	274	H	N	H	H	H	H	H	N	N
SNP4	539	G	A	G	G	G	G	A	A	A
SNP5	770	S	P	S	S	S	S	P	P	P
TMD1	Intron	□19	0	0	□19	0	0	0	0	0
Allotype		1	2	3	4	5	5	6	7	7
