# Supplementary material for: Homo-dimerization and ligand binding by the leucine-rich repeat domain at RHG1/RFS2 underlying resistance to two soybean pathogens
Source: BMC Plant Biol. 2013 Mar 15;13:43. doi: 10.1186/1471-2229-13-43 (PMC3626623; doi:10.1186/1471-2229-13-43)
Supplement: Additional file 7: Table S4 — Effect of three non-synonymous substitutions on protein stability calculated from the Fold X algorithm (http://fold-x.embl-heidelberg.de). The computed free energy of folding and the change in free energy between the wild type protein (Peking allele) and the mutant proteins is shown. The H to N and Q to K change increase the free energy of folding, whereas the alanine to valine change results in a significant decrease in the free energy. (DOC 45 kb) [file 1471-2229-13-43-S7.doc]

| Results of energy calculations | | |
| --- | --- | --- |
| PDB file | ΔG of folding (kcal/mol) | ΔΔG(kcal/mol) |
| Wild type | 70.25 | - |
| H274N | 70.67 | +0.42 |
| Q115K | 70.58 | +0.33 |
| A87V | 69.03 | −1.22 |
