# Supplementary material for: Homo-dimerization and ligand binding by the leucine-rich repeat domain at RHG1/RFS2 underlying resistance to two soybean pathogens
Source: BMC Plant Biol. 2013 Mar 15;13:43. doi: 10.1186/1471-2229-13-43 (PMC3626623; doi:10.1186/1471-2229-13-43)
Supplement: Additional file 8: Figure S4 — The 3D structures of LRR containing proteins showing high sequence homology to RHG1. Panel (a) shows the crystal structure of BRI1 (pdb id: 3RGX). Brassinosteroid recognition is mediated through the LRR domain of BRASSINOSTEROID-INSENSITIVE 1 (BRI1). BR1I exists as a monomer. The protein exists as a helical solenoid structure. (b) crystal structure of the Polygalacturonase inhibiting protein (PGIP). The PGIP protein (PDB id: 1ogq) is a cell wall localized protein that interacts with fungal endopolygalacturonases. (c) X-ray structure of the porcine ribonuclease inhibitor (PRI). The Leucine rich repeat of PRI (PDB id: 2BNH) forms tight complexes with ribonucleases thereby regulating RNA levels. PRI adopts a horseshoe configuration with the LRR motif composed of repeat beta-loop helix units. [file 1471-2229-13-43-S8.pptx]

## Slide 1
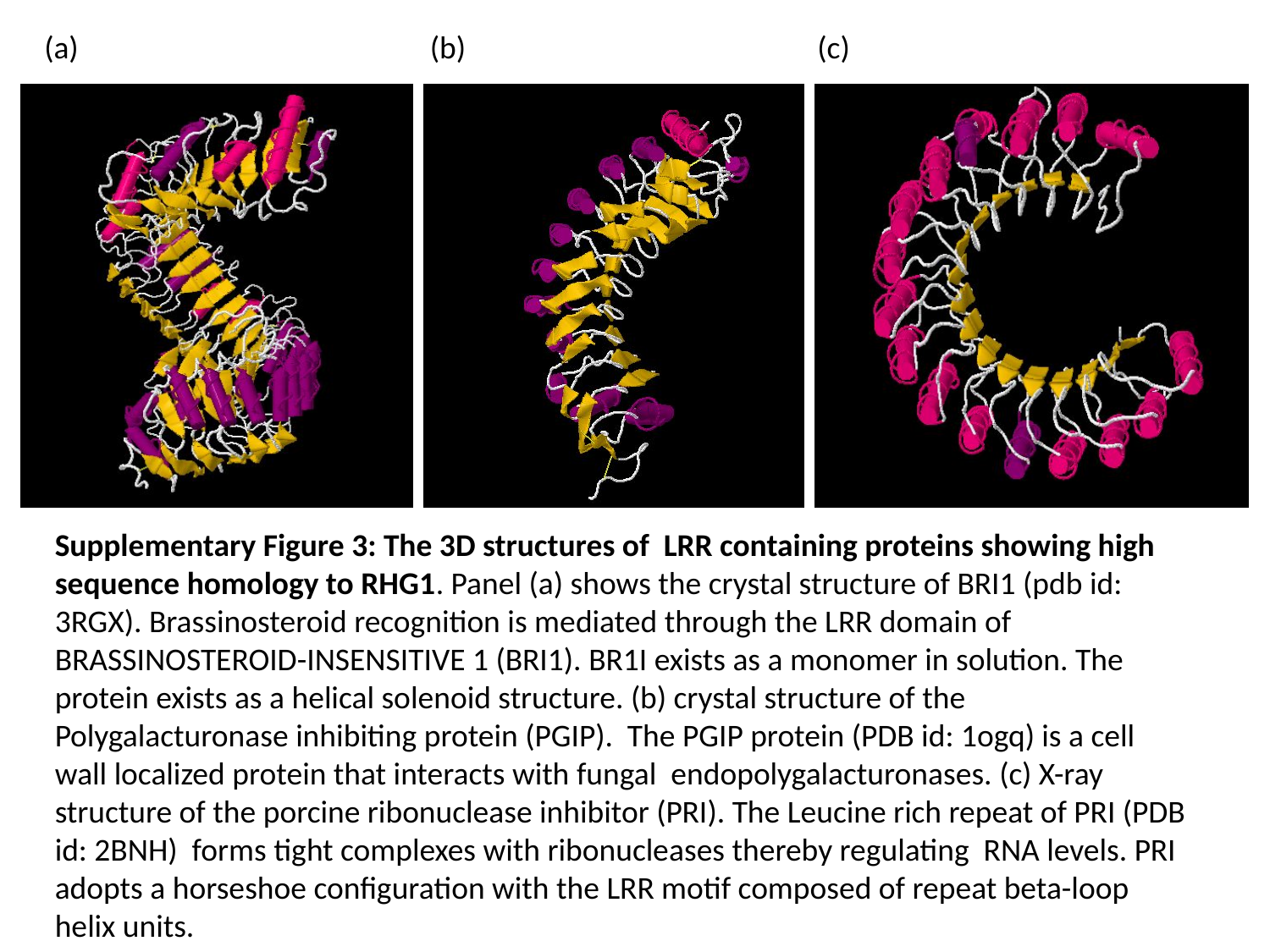

(a) (b) (c)
Supplementary Figure 3: The 3D structures of LRR containing proteins showing high sequence homology to RHG1. Panel (a) shows the crystal structure of BRI1 (pdb id: 3RGX). Brassinosteroid recognition is mediated through the LRR domain of BRASSINOSTEROID-INSENSITIVE 1 (BRI1). BR1I exists as a monomer in solution. The protein exists as a helical solenoid structure. (b) crystal structure of the Polygalacturonase inhibiting protein (PGIP). The PGIP protein (PDB id: 1ogq) is a cell wall localized protein that interacts with fungal endopolygalacturonases. (c) X-ray structure of the porcine ribonuclease inhibitor (PRI). The Leucine rich repeat of PRI (PDB id: 2BNH) forms tight complexes with ribonucleases thereby regulating RNA levels. PRI adopts a horseshoe configuration with the LRR motif composed of repeat beta-loop helix units.
